# Preserving nature: domestic thrift and techniques of conservation in early modern England

**DOI:** 10.1098/rsnr.2021.0029

**Published:** 2022-06-20

**Authors:** Simon Werrett

**Affiliations:** Department of Science & Technology Studies, University College London, Gower Street, London WC1E 6BT, UK

**Keywords:** domestic history, thrift, experiment, preservation, conservation, repairs, collecting, medicine

## Abstract

Against an assumption that conservation practices only became ‘scientific’ in the late nineteenth and early twentieth centuries, this essay shows how, on the contrary, preservation techniques in early modern England were an inspiration for new forms of scientific inquiry and knowledge. Following the framework of ‘thrifty science’, the essay demonstrates how the thrifty value of making use and extending the life of goods encouraged a variety of preservation practices, which some scholars identified as valuable resources for a new experimental philosophy. In practice, preserving techniques crossed between domestic, experimental and academic sites. Since ‘thrifty science’ included the preservation of human and non-human ‘bodies’, the essay argues that an appreciation of early modern conservation necessitates an interdisciplinary approach.

## Introduction

Prior to the mid-nineteenth century, broken chinaware could be repaired with rivets, a skilled procedure in which heated staples were inserted into holes in the pieces of a bowl or plate and allowed to cool, drawing the sections together to seal the crack. By the 1850s, however, a new class of restorers and conservators began to remove rivets from valuable china, making invisible seals that returned the china to its ‘original’ state. As Morwenna Blewett has shown, the rise of professional conservation and restoration in this period was hailed as a moment when the practice was said to have became ‘scientific’, a laboratory enterprise that superseded crude and amateur repairs.^1^

Extending the framework of my recent book *Thrifty science* to the history of conservation, this essay argues that practices of repair and preservation were by no means unscientific prior to the second half of the nineteenth century, at least in the context of seventeenth-century England.^2^ On the contrary, they played a significant role in the establishment of modern science itself. Preservation constituted one element of a routine enterprise of thrifty living in early modern households that was in fact constitutive of the new science of that era. Before the nineteenth century, most experimenting happened in the home.^3^ Domestic acts of preservation caught the attention of early modern scholars, who saw in them a form of practical knowledge that might serve as a replacement for what many considered the useless scholastic natural philosophy of the universities. What became the ‘experimental philosophy’, one of the key ingredients of modern science, found some of its inspiration in domestic acts of preserving and repairing possessions.

This essay describes the features of ‘thrifty’ household ‘oeconomy’ in early modern England and examines its distinctive approach to materials, issues of damage and deterioration, and the value of preserving things. It provides a broad context for some of the particular conservation practices relating to art and architecture discussed elsewhere in this issue. Thrift led to experimentation and acts of preservation both within the home and beyond its walls in museums and academies. Focusing on specific techniques of cleaning, repair and preservation I argue that, from the perspective of thrift, preservation techniques cut across a variety of modern divisions so that a history of conservation in this period will need to be both a social and material history of the household, a history of medicine and a history of science. I conclude with some brief considerations of why thrifty preservation, with its riveted china bowls, gave way to a professional culture of ‘restoration’ in the nineteenth century and came to appear as ‘unscientific’ from this later perspective.

## Thrift and experiments in preservation

‘Conservation’, ‘preservation’, ‘keeping’ (as in housekeeping) and ‘maintenance’ were terms used interchangeably to refer to the practices of upholding life, health or the good order of things in early modern England. The preservation of goods extended to a variety of enterprises, but here I confine my discussion to the home. Historians of science have given increasing attention to the home as a site of natural knowledge making, and the home is equally important for the history of conservation.^4^ While conservation is typically associated today with museums and collections, it formed a significant element of domestic activity within early modern homes. The ‘home’, of course, is a broad category and encompassed diverse social and architectural forms. Early modern literature on ‘oeconomy’ or household management was typically aimed at literate male householders managing a family and servants, ranging from a few persons to a large aristocratic estate. The home was not a clearly defined or fixed category but an open-ended space where a great diversity of medical, scientific, artisanal, collecting and domestic experiments were undertaken by both men and women. Homes were porous spaces, connected up with universities, academies, workshops, museums and other institutions in a dynamic manner, as bodies and skills moved routinely between them.

It makes little sense to distinguish the home too sharply from these sites. Robert Hooke, for example, the Royal Society's curator of experiments in the late seventeenth century, moved regularly between trying out experiments in his quarters and showing them in the Royal Society's formal meetings.^5^ Householders also collected such things as furnishings, clothing, ornaments, plants and art, which could shift in and out of more dedicated or distinct spaces over time. We read of Elias Ashmole, founder of the Ashmolean Museum in Oxford, that he ‘had lodged and boarded sometimes at a house in South Lambeth, kept by Mr. John Tradescant, whose father and himself … had collected a vast number of curiosities, which … Mr. Tradescant and his wife determined to bestow on Mr. Ashmole’ in 1659.^6^ Ashmole kept this ‘closet of curiosities’ in his home in London and then presented it to Oxford in 1682, to be located in a dedicated museum.^7^ This openness between different sites was not accidental but itself symptomatic of a thrifty culture, where the uses of things were often more important than any strict definitions of their boundaries and identities.

The literature on oeconomy or household management circulated in both print and manuscript in early modern England. Works ranged from family recipe books gathered over several generations to printed works written by experienced household patriarchs. Texts gave advice on managing the family and servants, keeping accounts and maintaining one's property and possessions in good order. Recipe books collected instructions for preparing food, medicine, cleaning and repairs.^8^ Householders also read books of secrets on artisanal techniques.^9^ These works might be aimed at husbands (a medieval term meaning ‘house-bound’) or housewives, and concerned husbandry (farming, estate management) or housewifery (cookery, cleaning, care of the family, accounts) accordingly. In practice, both sexes undertook labours associated with particular genders in print, again indicating a fluidity in domestic boundaries.

One of the values that oeconomic works promoted was *thrift* or *frugality*, terms that were used interchangeably in the period. Today thrift means saving money but in early modern England it had a different meaning, centred on finding a balance between spending and saving, between buying new and making use of what one already owned. As the anonymous *The Art of Thriving* advised householders in 1674, ‘Spare not, nor spend too much, be this thy care.’^10^ Thrift encouraged consumption, but also reuse. While the history of consumption has received great attention from historians, there has been less appreciation of how early modern people made use of what they already had, even though this was a commonplace practice.^11^

Thrift thus encouraged householders to find new uses for things, a practice called ‘making use’ or ‘putting into service’. One constantly sees in this era householders exploring how material things could be used for new purposes. Straw or feathers did ‘service’ as padding inside a mattress; old garments were ‘altered’ into new ones; scraps were fed to domestic animals; feathers and breadcrumbs served to clean paintings; recipes might be varied by adding leftovers from previous meals.^12^ Similarly, there was much concern to preserve things, so that their serviceable lives could be extended as long as possible. In *The Gentleman's Calling* (1660), the churchman and provost of Eton Richard Allestree advised his readers on the ‘well husbanding’ and ‘prudent managery’ of their households, which entailed ‘having such a provident care of those goods, and possessions … as may secure them from that Consumption, to which carelessness and sloth will infallibly betray them’.^13^

Householders spent much time on these acts of care: cleaning, repairing and reworking things to ensure that they endured. These endeavours lent domestic life a distinctively creative and experimental character, as householders tried out new techniques, recipes and methods, routinely varying ingredients and the uses of things. The term ‘experiment’ had a broad meaning in early modern England and was applied by male and female householders to various domestic enterprises. The seventeenth-century writer on cookery and female domestic duties Hannah Woolley entitled one of her books *Choice Experiments & Curiosities of Preserving in Jellies and Candying both Fruits & Flowers*.^14^ Medical and culinary recipes were referred to as experiments, and tested by householders, who recorded their success or failure in manuscript notebooks.^15^

## The science of thrift

Preservation thus formed part of an ongoing creative and innovative enterprise among householders. This was by no means a ‘pre-scientific’ form of conservation, but played a role in shaping new scientific methods and knowledge. Most experimentation happened in the home. Natural philosophers saw in domestic preservation an inspiration for ‘experimental philosophy’, a form of inquiry that would surpass the sterile scholasticism of university learning. As Francis Bacon wrote in his works promoting experiment as a new method in science,

The preparation of meats, bread, and drinks, if it be well ordered and agreeable to this intention [the progress of natural knowledge], is of very great importance. And although it be a thing mechanical and savouring of the kitchen and the cellar, yet it is worth more than the fables of gold, precious stones, and the like.^16^

Bacon died after catching a cold while experimenting on preservation. Arguing with a companion, Dr Witherborne, he insisted that freezing would preserve fresh meat. They obtained a chicken, from a ‘poore womans howse’, gutted it and packed it with snow and buried it to find out. But Bacon was promptly taken ill with ‘such a cold that in 2 or 3 days … he dyed of Suffocation’.^17^

Later in the seventeenth century the Irish chymist Robert Boyle (described by John Aubrey as ‘very temperate, and vertuouse, and frugall’) argued that ‘making use’ was fundamental to what he now called the ‘experimental philosophy’.^18^ In an essay on ‘men's great ignorance of the uses of natural things’ he noted that ‘there is scarce any one thing in nature wherof the uses to human life are yet thoroughly understood’.^19^ Boyle discussed various forms of preservation in the essay, including the prevention of rust using oil and the varnishing of wood to keep out damp.

Everyday acts of preservation and repair were thus by no means unscientific, but rather were considered as valuable resources for a new science. Thrift also shaped practices of collecting. In a late-eighteenth-century pamphlet, under the motto ‘Let nothing be lost!’ Thomas Butterworth Bayley explained his thoughts ‘on the necessity and advantages of care and oeconomy in collecting and preserving different substances for manure’. He gave detailed instructions to estate owners on the collection of different kinds of mud, urine, coal ashes and street sweepings for making manure, and explained how to preserve them in reservoirs and dunghills. Meanwhile, scholars collected letters and papers to preserve them for posterity. Robert Stephens published Francis Bacon's letters in 1702, motivated by ‘the desire I had to preserve the least Remains of this Noble Lord from the Fate incident to loose Papers’.^20^

Thrifty values might cut across the home and new academic institutions. Collections passed between private homes and institutions to serve the goal of ‘making use’. In the middle decades of the seventeenth century, the music master Robert Hubert formed a private cabinet of curiosities whose catalogue noted the many uses of its contents. A swordfish from the Black Sea, for instance, was ‘excellent meat sliced and broyled, with oyle, pepper, salt, and the juice of a lemon on it.’^21^ Other items were notable for the diversity of their uses, such as ‘A Cocos fruit whole; the fruit and tree affords many necessary things for the benefit of Man, as milk, wine, water, oyle, needles, threed, boards, cordage, sayles, and other things’.^22^ In the 1660s the Royal Society purchased Hubert's collection as the basis of its repository. Nehemiah Grew, who curated it, also viewed collecting as an occasion for making bodies more serviceable, writing of his catalogue of the repository that ‘in such Descriptions, many Particulars relating to the Nature and Use of Things, will occur to the Authors mind, which otherwise he would never have thought of. And may give occasion to his Readers, for the consideration of many more’.^23^ Bringing things together into a textual or physical space was an opportunity for better understanding how to multiply service. Like Hubert, Grew recorded examples in the collection, such as the long palmeto leaf, ‘which both the *Arabians* and *Indians* make use of to write upon, by *Impression* with a *Style*’.^24^

## Preserving bodies

Understanding the domestic culture of preservation thus requires consideration of both the history of domestic oeconomy and the history of science, since practices and personnel regularly moved between the home and scientific institutions. This also requires an appreciation of the history of medicine because household thrift applied not just to domestic goods like furniture or foodstuffs but also to the people who made up the household: thrift was about caring for the family and servants in addition to material possessions. It is notable that early moderns did not speak of ‘objects’ or ‘subjects’ but rather of ‘bodies’, encompassing both humans and non-humans.^25^ Ashmole called the items in his collection ‘bodies’ and it was a common term for both people and things. Preservation work incorporated the medical treatment of persons to ensure that, like other bodies, they should endure as long as possible. This proximity between human and non-human bodies is apparent in the language used to describe bodily preservation. ‘[H]ealth implies only keeping the machine of the body in perfect order’, explained one eighteenth-century writer; ‘for the prolonging of life, great care must be taken to preserve the materials, that they may be as little worn as possible, and consequently last as long as the nature of them will allow’.^26^

It was quite unusual to refer to ‘objects’ in early modern England as being ‘damaged’ and more common to speak of ‘bodies’ that were ‘injured’, ‘offended’, ‘bruised’, ‘suffering’ or ‘deranged’, all of which terms might also apply to people. Faced with injured goods (or ‘casualties’), householders might seek their ‘recovery’. A recipe in an eighteenth-century commonplace book now in the Wellcome Collection describes ‘A Bitum[en] lute to cure Crackt Jarres as Stone Carvers use’.^27^ Notice that it speaks of ‘curing’ cracked jars, as if they are ill. Artisans used similarly animate language to describe material objects. The printer Joseph Moxon claimed that ‘the force that must injure a Tennant [joint], must offend it [a]cross the grain of the wood, in which position it will best indure violence’.^28^ The term ‘service’ also captured this common talk related to bodies. Both material and human bodies could do service and be servants in a household. From our perspective, just as inanimate objects might be discussed like human bodies, so humans might be spoken of, and treated, more like inanimate objects. It was common to see the body as a machine and, in practices of slavery and servitude, human bodies were reduced to the status of objects.^29^

Thrift thus entailed simultaneously a material and a moral way of talking about bodies. Early moderns understood the identities and fates of humans and non-humans as intimately bound up together. Both Ashmole and Grew played on this conflation when writing about collections. Verses opening Ashmole's *Musaeum Tradescantianum* of 1656 praised John Tradescant the Younger as ‘Heire of thy Fathers goods, and his good parts / Which both preservest, & augment'st his store’.^30^ ‘Goods’ were moral and material, stored up in people as much as collections. Similarly, Grew linked the creation of the Royal Society's repository with a moral and material restoration, in praise of the patron of the collection, Daniel Colwall. Since the Royal Society was ‘seeming … to look a little pale’, he said, ‘you intended hereby, to put some fresh Blood into their Cheeks; pouring out your Box of Oyntment, not in order to their Burial, but their Resurrection’.^31^

The glue that bound together the material and the moral in this culture was religion. While current definitions of thrift associate it principally with an economic imperative to save money, the early modern English viewed thrift as a Christian virtue. To be thrifty led to ‘thriving’, a closely related term, and to thrive was to make the most of the material resources that God had provided.^32^ As the Puritan Richard Baxter wrote in his *Christian Directory* of 1673, ‘We must see that nothing of any use be lost through satiety, negligence, or contempt; for the smallest part is of God's gifts and talents, given us, not to cast away, but to use as he would have us.’ As such, thrift cut across classes. Certainly the poor were obliged to be sparing and the court and aristocracy were expected to display wealth, but thrift was still a desirable trait for any Christian.^33^ Preservative Christian morals were engraved into everyday life. As Tara Hamling and Catherine Richardson have shown, seventeenth-century English houses were routinely decorated with biblical quotations and carvings intended to secure householders from physical peril and from sin, so that the house was a site of both material and moral decay and redemption.^34^ ‘I can shew you a man's character in his house’, wrote the lawyer Roger North in 1698. ‘If he hath bin given to parsimony or profusion … his edifices shall be tincted accordingly, and the justness or imperfections of his mind will appear in them.’^35^ Experiments in making use helped make manifest this moral character, though no doubt householders’ actual ability to live up to ideals of moral and material preservation varied enormously. John Aubrey recorded of the physician William Harvey that ‘For twenty years before he died he took no manner of care about his worldly concerns, but his brother Eliab, who was a very wise and prudent manager, ordered all … faithfully.’^36^ Nevertheless, there is sufficient evidence of thrifty practices to suppose that moral directives did have some effect.

## The techniques of preservation

Early modern English householders were thus expected to take great care in preserving their ‘goods’ from physical and moral corruption. Not everything needed caring for, of course—witness the abundance of oyster shells and clay pipes that wash up in their thousands on the Thames foreshore, or the broken crucibles and animal bones thrown on the dust heap that were excavated from the site of the Old Ashmolean Museum in Oxford in 1999.^37^ These were the ‘disposable’ goods of early modern England. But householders deployed a wide variety of techniques to ensure preservation. These ranged from careful storage and use, cleaning and maintenance, to repairs if bodies became ‘injured’, or, if something was not recoverable, reuse and recycling.

Goods were often designed to be durable, by keeping their forms simple, by making their parts accessible for repair and by employing materials that were secure from corrosion and decay. In 1666 Robert Boyle proposed improvements to the baroscope which included replacing steel parts with copper and brass, since these were ‘less subject, than Steel … to rust with long standing’. A beam could be lightened to avoid stressing it and the whole instrument might be encased in a glass case to inhibit dust and ‘irregular agitations’. The large glass ‘bubble’ used in the instrument was particularly prone to injury, so Boyle suggested using two smaller ones to avoid ‘the difficulties and casualties, that may happen about the procuring and preserving such large and light Bubles’.^38^

In collections of medical recipes, authors described how to make medicaments with a remarkably long shelf-life. The Countess of Kent reckoned her leaden plaster would ‘keep twenty years, the older the better’.^39^ The recipe book of the wealthy householder Mary Granville described how to make a water with green walnuts for dropsies and palsies, ‘distill them in a Glasse-still & keep it for your life’.^40^ Other goods were reworked to keep them up to date, as in the practice of ‘clobbering’ blue and white Chinese export porcelain by painting more fashionable decorations over the original ornaments.^41^ As Anna Marie Roos has shown, copperplates used for scientific illustrations were also reused and reworked to keep them up to date, and painters retouched or overdrew canvases to the same end.^42^

To keep bodies safe from injury, homes typically contained an assortment of storage vessels or ‘receptacles’, ranging from chests and trunks for clothes or curiosities to glass frames for protecting pictures.^43^ Robert Hubert noted of his collection, ‘there are in a Chest great variety of strange Bones, Teeth and Clawes of many different Creatures’ and ‘many hundreds of … beautifull shells of fishes … in chests and boxes’.^44^ Householders made use of existing possessions to improvise storage. Prints were fixed into albums using sealing wax.^45^ Botanists turned books into containers by inserting pages holding dried specimens.^46^ Johann Dillenius, a German who spent the 1720s and 1730s botanizing in England and Wales, mounted his collection of dried plants in a herbarium of paper sheets surrounded with segments of wallpaper that were evidently ready to hand. Seeds were wrapped up in old letters and used envelopes.^47^ Naturalists created archives in their homes to preserve correspondence and collections for the next generation.^48^ John Tradescant made use of his house as a location for the collections he amassed as an employee of the Duke of Buckingham and King Charles I.

The home itself was a container for keeping bodies secure. Positioning bodies in different rooms gave different kinds and degrees of protection. Tradescant's house's open courtyard held ‘two ribs of a whale’, which presumably would have not degraded with the weather, while smaller, more delicate items were kept indoors: ‘In the museum itself we saw a salamander, a chameleon, a pelican, a remora, a lanhado from Africa, a white partridge, a goose.’^49^ Houses were liable to their own risks, however, particularly fire, flooding and theft. Ashmole was much concerned with theft. Inventories counteracted the problem. After winning ownership of the Tradescant collections, Ashmole was obliged to wait until Hester Tradescant's death before taking possession of them. The court managing the process appointed a commission to ensure that the collections were not subject to ‘Spoyle & Imbezellm[e]nt’ by periodically checking them against a catalogue Ashmole drew up in 1656.^50^ Later, when Ashmole proposed statutes for the Ashmolean Museum in Oxford, he insisted on similar visitations to deter the ‘deteriorating of my donation’.^51^

Images also captured and preserved the state of goods at a particular time. Households kept prints, paintings and portraits to record ancestors, estates, pets, possessions or significant episodes of family history.^52^ Art resisted loss. Ashmole's statutes explained,

That whatsoever naturall Body that is very rare, whether Birds, Insects, Fishes or the like, apt to putrefie … decay with tyme, shalbe painted in a faire Velome Folio Booke, either with water colors, or at least design'd in black … white, by some good Master, with reference to the description of the Body itselfe … the mention of the Donor, in the Catalogue; which Booke shalbe in the Custody of the Keeper of the Musaeum, under Lock … Key.^53^

Preservation through painting assumed some degree of *Ruinenlust*, or an acceptance of decay and deterioration in things. Bodies could only be preserved for so long, and it was another feature of early modern homes and collections that they contained broken goods that served for decoration or were retained even when they no longer functioned.^54^

Careful handling was another means to preserve bodies in good order. Servants were routinely warned in books of household management to take care not to break their masters’ things. A servant should be ‘as careful and frugal of [their master's] property as they would be of their own’.^55^ ‘Conveyance’—transporting goods—also carried risks, and householders might avoid moving something if they thought it could be injured in the process. ‘Concerning the new booke’, explained Bruno Ryves to Ashmole in 1669, ‘I had no conveniency of safe conueyance to you, therfor deferre the sending of it, meaning (God willing) shortly to bring it my selfe.’^56^

Another way to avoid injuries was by cleaning, which was important maintenance work for any home or institution. After Ashmole's collections were transferred to Oxford he stipulated that they be regularly cleaned, explaining in the statutes that the ‘Keeper shall allow a person, to sweepe … clense the Musaeum … Closetts, with such other things therein preserved, as he shall appoint, a reward not less then Forty shillings a yeare’.^57^ Numerous early modern works gave advice on how to clean glass, silver-plate, lace, jewellery, stains in silk and wool, furniture and marble, all involving a wide range of skills, techniques and materials. Eliza Haywood, author of a book on the conduct of serving maids, provided recipes for cleaning everything from pimples on the face to oily spots on parchments.^58^ Techniques applied to bodies that today might be divided between the history of art, the history of medicine and domestic history. Laundry, for example, was cleaned using various ingredients such as lemon juice, burned and powdered sheep bones, and solutions of lye and ashes, which could also be used to remove stains and spots from old prints.^59^ Robert Boyle's recommendations for washing an air-pump used solutions commonly seen in recipe books of the time.^60^ Laundering clothes also inspired experiment. Housewives, Bacon noted in one of his manifestos for experiment, knew from the ‘Laundry of Cloaths’ what the different qualities of water were: chalky water would wear out garments and fatty water could best carry soap.^61^

Another important area of preservation in the home concerned food. Cookery books often contained recipes for the recovery of spoiled meat, bread and other dishes. Food was preserved from decay through salting, pickling, smoking and drying, while fruit was boiled with sugar to make candies, preserves and conserves.^62^ The author of *The Compleat Cook* (1694) explained how to cook venison fast, ‘in a quick and frugal way’, and described how to recover ‘tainted’ meat so that it was not wasted: ‘Take your Venison and lay it in a clean cloth, then put it under ground a whole night, and it will remove the corruption, stink or savour.’^63^ Householders interested in botany and anatomy used rum, brandy and other spirits to preserve specimens, and the home was also a site for taxidermied hunting trophies and collections of animal bodies. Salt, pepper, alum and snuff were among the materials recommended to preserve them.^64^

This is only an indicative selection of domestic preservation techniques. The focus so far has been on ‘goods’ but the preservation of human bodies was equally important, and families routinely prepared or purchased medications for use in the home.^65^ Clothing was maintained through a rich culture of alterations and reworking by seamstresses and tailors. Buildings, estates and livestock were kept in good order using still more techniques.^66^ The same high level of attention was given to repairing goods as keeping them safe and clean, once again in order to extend their serviceable lives. Repair skills ranged from what were called ‘shifts’ or improvisations which cobbled together a solution on the hoof, to highly elaborate artisanal work, undertaken by itinerant chairmenders and tinkers or clockmakers and optical and mathematical instrument makers who might set up business in a town. Alexi Baker and Richard Dunn have examined instrument repairs, showing how international networks of skilled makers grew up around ports and trading cities like London to repair telescopes, navigational instruments, timekeepers and barometers. The work was meticulous and could take from days to several years, involving specialized techniques such as ‘touching’ or remagnetizing compass needles.^67^ Once again, because householders shifted between enacting repairs themselves and taking injured goods to specialists, the home was always connected to diverse external sites of expertise and skill.

## Conclusion

To conclude, three general observations regarding thrift and preservation may be made. First, it is notable that the prevention of injury was a critical means of preservation in homes and other early modern institutions. There were as many stipulations for avoiding injury as for remedying it, and a history of early modern conservation should keep this in mind, as a focus only on techniques of repair and restoration would be a distortion. The threat of fire, for example, was countered with diverse practices ranging from prayers to the removal of goods. When the Great Fire threatened London in 1666, Elias Ashmole transported his books from the Temple to Lambeth to avoid the flames, though only to see the returned collection consumed by another fire in January 1679.^68^ Ideally, household oeconomy, like good regimen of diet, would keep bodies in a state of good health before they could suffer any injury.

Second, preservation techniques were invariably social as well as material endeavours. Just as they mixed the material and the moral, so practices like cleaning and storage invariably enrolled diverse human and non-human actors. Preservation happened at the intersection of bodies, involving families, friends, neighbours and communities, working collectively or exchanging recipes or techniques. Bodily identities were forged, negotiated and reworked in the process. Different expectations of gender, class and race determined who could clean, repair or preserve what.^69^ Women might be tasked with domestic drudgery like laundering clothes while, in ports and cities, male instrument makers offered their services repairing clocks, barometers and scientific instruments. Identities were shaped and transformed in acts of preservation: a ‘washerwoman’ only became such when she engaged with the material paraphernalia of cleaning linen.

Third, acts of preservation were also acts of innovation, so that preservation was not about stasis or the end of a body's serviceable life, but constituted a dynamic and creative enterprise. To return to the example of china riveting, there was constant experimentation in the eighteenth century to devise more durable china or better sealants and glues to mend cracks and breaks in vessels. Artisans and householders took pride in this work and, as Sara Pennell has noted, this manifested in visible signs of repairs and preservation being kept or even displayed on goods.^70^ China rivets, the signatures of repairers and substitute parts made from different materials all spoke of the efforts of owners to prolong the lives of their possessions, a sign ultimately of Christian virtue.

The rise of a ‘scientific’ conservation culture in the nineteenth and early twentieth centuries, as Pennell also notes, ‘complicated the visibility of historic repairs’, since it gave value to more invisible mending and the removal of imperfections in displayed objects.^71^ In *Thrifty science*, I noted how diverse nineteenth-century contexts including industrialization, mechanization, and the rise of a more independent and professional scientific community coincided with a radical shift in attitudes towards domestic thrift and cultures of repair and preservation. Household practices were downgraded to the status of amateur tinkering in comparison with a supposedly more professional scientific and industrial technique.^72^ In this context, ‘men of science’ sought to distance themselves from domestic tradition, promoting expensive, specialized and professionalized skills over what they now represented as homely improvisations. Perhaps the shift to ‘scientific’ conservation followed a similar path, eschewing thrifty domestic preservation techniques in order to carve out a distinct, professional identity and practice. Recognizing this may open up to historical scrutiny a wider range of early modern preservation techniques and different ways of thinking about repairs and restoration.

## Data Availability

This article has no additional data.

## Footnotes

1 Isabelle Garachon, ‘Old repairs of china and glass’, *Rijksmuseum Bull.* **58**, 35–54 (2010); Emily Cockayne, *Rummage: a history of the things we have reused, recycled and refused to let go* (Profile, London, 2020), pp. 221–223; Harold J. Plenderleith, ‘A history of conservation’, *Stud. Conserv.* **43**, 129–143 (1998); Vincent Daniels (ed.), *Early advances in conservation* (British Museum, London, 1988); Ellen Pearlstein, ‘Conservation and preservation of museum objects’, in *Encyclopedia of library and information sciences* (ed. John D. McDonald and Michael Levine-Clark), 4th edn, pp. 1068–1075 (Taylor & Francis, London, 2017); Morwenna Blewett, Introduction to this special issue.

2 Simon Werrett, *Thrifty science: making the most of materials in the history of experiment* (University of Chicago Press, Chicago, 2019).

3 Steven Shapin, ‘The house of experiment in seventeenth-century England’, *Isis* **79**, 373–404 (1988).

4 Alix Cooper, ‘Home and household as sites for early modern science’, in *The Cambridge history of early modern science* (ed. Katharine Park and Lorraine Daston), pp. 226–237 (Cambridge University Press, New York, 2006); Simon Werrett, ‘Household oeconomy and chemical inquiry’, in *Compound histories: materials, production, governance, 1760–1840* (ed. Lissa Roberts and Simon Werrett), pp. 35–56 (Brill, Leiden, 2017); Donald L. Opitz, Staffan Bergwik and Brigitte Van Tiggelen (eds), *Domesticity in the making of modern science* (Palgrave Macmillan, Basingstoke, 2016); Deborah R. Coen, ‘The common world: histories of science and domestic intimacy’, *Mod. Intellect. Hist.* **11**, 417–438 (2014).

5 Shapin, *op. cit.* (note 3).

6 G. R. Corner, ‘Elias Ashmole, his house and lands at South Lambeth’, *Surrey Archaeol. Collect.* **2**, 17–26 (1864). See also Marjorie Swann, *Curiosities and texts: the culture of collecting in early modern England* (University of Pennsylvania Press, Philadelphia, 2001), pp. 46–48.

7 Corner, *op. cit.* (note 6), p. 23.

8 Karen Harvey, *The little republic: masculinity and domestic authority in eighteenth-century Britain* (Oxford University Press, Oxford, 2012); Elaine Leong, *Recipes and everyday knowledge: medicine, science and the household in early modern England* (University of Chicago Press, Chicago, 2018); Wendy Wall, *Recipes for thought: knowledge and taste in the early modern kitchen* (University of Pennsylvania Press, Philadelphia, 2015); Werrett, *op. cit.* (note 2), pp. 17–18.

9 Allison Kavey, *Books of secrets: natural philosophy in England, 1550–1600* (University of Illinois Press, Champaign, IL, 2010).

10 Anon., *The Art of Thriving* (London, 1674)*,* p. 1; Werrett, *op. cit.* (note 2), pp. 20–26.

11 The exception is the study of early modern second-hand circulations but I would argue that this was only one element of a broader thrifty culture. See e.g. Jon Stobart and Ilja Van Damme (eds), *Modernity and the second-hand trade: European consumption cultures and practices, 1700–1900* (Palgrave Macmillan, Basingstoke, 2010).

12 Werrett, *op. cit.* (note 2), pp. 26–29; Alan Donnithorne, ‘Early approaches to the conservation of works of art on paper: cleaning, repair and restoration’, in Daniels, *op. cit.* (note 1), pp. 15–25, at p. 16. Anne Goldgar and Miles Ogborn note the deliberate concern of collectors in early modern Europe for the usefulness of their collections: Anne Goldgar and Miles Ogborn, ‘Introduction: early modern collections in use’, *J. Hist. Collect.* 1–6 (2021), at p. 2 (https://doi.org/10.1093/jhc/fhab013).

13 Richard Allestree, *The Gentleman's Calling* (London, 1660), p. 46.

14 Hannah Woolley, *The Ladies Directory in Choice Experiments & Curiosities of Preserving in Jellies and Candying both Fruits & Flowers* (London, 1662).

15 Simon Werrett, ‘Food, thrift, and experiment in early modern England’, *Glob. Food Hist.* 1–17 (2021), at p. 4 (https://doi.org/10.1080/20549547.2021.1942666); Leong, *op. cit.* (note 8), p. 101.

16 Francis Bacon, quoted in Werrett, *op. cit.* (note 2), p. 51.

17 John Aubrey, *Brief lives* (ed. Kate Bennett), 2 vols (Oxford University Press, Oxford, 2018), vol. 1, pp. 210–211.

18 *Ibid.*, vol. 1, p. 53; Werrett, *op. cit.* (note 2), p. 38.

19 Robert Boyle, *The works of the honourable Robert Boyle* (ed. Thomas Birch), 6 vols (London, 1772), pp. 470–494, at p. 470.

20 Thomas Butterworth Bayley, *Thoughts on the necessity and advantages of care and oeconomy in collecting and preserving different substances for manure*, 3rd edn (C. Wheeler & Son, Manchester, 1799); ‘Preface’, in Francis Bacon, *Letters of Sr Francis Bacon, Baron of Verulam, Viscount St. Alban, and Lord High Chancellor of England. Written during the reign of King James the First* (ed. Robert Stephens), unpaginated (London, 1702).

21 Robert Hubert, *A catalogue of many natural rarities with great industry, cost, and thirty years travel in foraign countries collected by Robert Hubert, alias Forges* (London, 1665), p. 27. Other species yielding good meat included the sea-mouse (p. 17), soldier-fish (p. 19) and armadillo (p. 35).

22 *Ibid.*, p. 46.

23 Nehemiah Grew, *Musæum regalis societatis, or, a catalogue & description of the natural and artificial rarities belonging to the Royal Society and preserved at Gresham College* (London, 1681), ‘Preface’.

24 *Ibid.*, p. 184.

25 The best-known exponent of these terms is Bruno Latour. See Colin Barron, ‘A strong distinction between humans and non-humans is no longer required for research purposes: a debate between Bruno Latour and Steve Fuller’, *Hist. Hum. Sci.* **16**, 77–99 (2003).

26 Johann Heinrich Cohausen, *Hermippus redivivus: or, the sage's triumph over old age and the grave* (London, 1771), p. 97.

27 Boyle Godfrey's commonplace book, *ca* 1740, MS 2534, f. 675, Wellcome Collection, London. Thanks to Marie Thébaud-Sorger for this reference.

28 Joseph Moxon, *Mechanick exercises, or, the doctrine of handy-works*, 2nd edn (London, 1693), p. 81; Werrett, *op. cit.* (note 2), p. 98. See also Pamela H. Smith, *The body of the artisan: art and experience in the Scientific Revolution* (University of Chicago Press, Chicago, 2004).

29 Sara D. Schotland, ‘Africans as objects: Hogarth's complex portrayal of exploitation’, *J. Afr. Am. Stud.* **13**, 147–163 (2009).

30 Gualterus Stonehousus, ‘To John Tradescant the younger, surviving’, in John Tradescant, *Musæum Tradescantianum: or, a collection of rarities. preserved at South-Lambeth neer London* (London, 1656), unpaginated.

31 Grew, *op. cit.* (note 23), ‘Epistle dedicatory’.

32 In a Spanish context, Constanza Beltrami discusses in this special issue how, in the construction of Segovia Cathedral, the recycling of existing building materials saved neither time nor money, but the work was considered redemptive for the Catholic subjects who carried it out.

33 Richard Baxter, *A Christian Directory* (London, 1673), p. 146; Werrett, *op. cit.* (note 2), p. 27.

34 Tara Hamling and Catherine Richardson, *A day at home in early modern England: material culture and domestic life, 1500–1700* (Yale University Press, New Haven and London, 2017), pp. 44–45.

35 Roger North, quoted in Colin Pratt, *The great rebuildings of Tudor and Stuart England: revolutions in architectural taste* (Routledge, London and New York, 1994), p. 29.

36 John Aubrey, quoted in Werrett, *op. cit.* (note 2), p. 40.

37 Ted Sandling, *London in fragments: a mudlark's treasures* (Frances Lincoln, London, 2016); J. A. Bennett, S. A. Johnston and A. V. Simcock, *Solomon's house in Oxford: new finds from the first museum* (Museum of the History of Science, Oxford, 2000).

38 Robert Boyle, ‘An account of a new kind of baroscope, which may be called statical; and of some advantages and conveniencies it hath above the mercurial’. *Phil. Trans. R. Soc. Lond.* **1**, 231–239 (1665–1666), at pp. 235–236.

39 Elizabeth Grey, Countess of Kent, *A choice manual, or rare secrets in physick* (London, 1687), p. 106.

40 Mary Granville, quoted in Kristine Kowalchuk (ed.), *Preserving on paper: seventeenth-century Englishwomen's receipt books* (University of Toronto Press, Toronto, 2017), p. 138.

41 Helen Espir, *European decoration on oriental porcelain, 1700–1830* (Jorge Welsh, London and Lisbon, 2005).

42 Anna Marie Roos, ‘The art of science: a “rediscovery” of the Lister copperplates’, *Notes Rec. R. Soc.* **66**, 19–40 (2012); Donnithorne, *op. cit.* (note 12), p. 20.

43 Catherine Hicks, ‘Early approaches to the conservation of works of art on paper’, in Daniels, *op. cit.* (note 1), pp. 7–14, at p. 9.

44 Hubert, *op. cit.* (note 21), pp. 7, 32.

45 Hicks, *op. cit.* (note 43), p. 7.

46 Anne Secord, ‘Containers and collections’, in *Worlds of natural history* (ed. Helen Curry, Nicholas Jardine, James Secord and Emma Spary), pp. 289–303 (Cambridge University Press, Cambridge, 2018), p. 296.

47 ‘Historia Muscorum’, website of the Oxford University Department of Plant Sciences, https://herbaria.plants.ox.ac.uk/bol/historiamuscorum (accessed 20 July 2021).

48 Elizabeth Yale, *Sociable knowledge: natural history and the nation in early modern Britain* (University of Pennsylvania Press, Philadelphia, 2016).

49 German visitor to Lambeth Georg Christoph Stirn, quoted in Swann, *op. cit.* (note 6), pp. 28–29, at p. 28.

50 Swann, *op. cit.* (note 6), pp. 47–48.

51 Elias Ashmole, ‘Statutes, Orders … rules, for the Ashmolean Museum, in the University of Oxford’ (1684), in C. H. Josten (ed.), *Elias Ashmole: his autobiographical and historical notes, his correspondence, and other contemporary sources relating to his life and work*, *vol. 4: texts 1673–1701*, pp.* *1821–1825 (Oxford University Press, Oxford, 1967), at p. 1822.

52 Robert Tittler, *Portraits, painters, and publics in provincial England 1540–1640* (Oxford University Press, Oxford, 2013).

53 Josten, *op. cit.* (note 51), p. 1823.

54 Werrett, *op. cit.* (note 2), p. 112.

55 Anon., *Domestic management; or the art of conducting a family* (London, 1800), p. 11.

56 Bruno Ryves, Letter to Elias Ashmole, 6 January 1669, in C. H. Josten (ed.), *Elias Ashmole: his autobiographical and historical notes*, *vol. 3: texts 1661–1672*, pp. 1154–1156 (Oxford University Press, Oxford, 1967), at p. 1155.

57 Josten, *op. cit.* (note 51), p. 1824.

58 Eliza Haywood, *A new present for a serving maid* (London, 1771), pp. 248–268.

59 Drea Leed, ‘“Ye shall have it cleane”: textile cleaning techniques in renaissance Europe’, *Medieval Clothing and Textiles* **2**, 101–119 (2006); Donnithorne, *op. cit.* (note 12), p. 17.

60 Werrett, *op. cit.* (note 2), p. 89.

61 Francis Bacon, *Sylua syluarum: or a naturall historie in ten centuries* (London, 1627), p. 104.

62 Lucy Havard, ‘“Preserve or perish”: food preservation practices in the early modern kitchen’, *Notes Rec. R. Soc.* **74**, 5–33 (2020); Stuart Thorne, *The history of food preservation* (Parthenon Publishing, Kirkby Lonsdale, 1986); C. Anne Wilson (ed.), *Waste not, want not: food preservation in Britain* (Edinburgh University Press, Edinburgh, 1991).

63 Anon., *The Compleat Cook; or, the whole art of cookery* (London, 1694), pp. 37–38.

64 Anita Guerrini, ‘The ghastly kitchen’, *Hist. Sci.* **54**, 71–97 (2016); Harold Cook, ‘Time's bodies: crafting the preparation and preservation of naturalia’, in *Merchants and marvels: commerce, science, and art in early modern Europe* (ed. Paula Findlen and Pamela Smith), pp. 237–247 (Routledge, London, 2002); Paul Lawrence Farber, ‘The development of taxidermy and the history of ornithology’, *Isis* **68**, 550–566 (1977); Anne Larsen, ‘Equipment for the field’, in *Cultures of natural history* (ed. N. Jardine, J. Secord and E. C. Spary), pp. 358–377 (Cambridge University Press, Cambridge, 1996); Dániel Margócsy, *Commercial visions: science, trade, and visual culture in the Dutch golden age* (University of Chicago Press, Chicago, 2014); see also Caitlin Play's contribution to this issue.

65 Anne Stobart, *Household medicine in seventeenth-century England* (Bloomsbury Academic, New York, 2016); Elaine Leong, ‘Making medicines in the early modern household’, *Bull. Hist. Med.* **82**, 145–168 (2008); Elaine Leong, ‘“Collecting knowledge for the family”: recipes, gender and practical knowledge in the early modern English household’, *Centaurus* **55**, 81–103 (2013).

66 On clothing, see Cockayne, *op. cit.* (note 1), pp. 222–223; on livestock, see Louise Hill Curth, *The care of brute beasts: a social and cultural study of veterinary medicine in early modern England* (Brill, Leiden, 2010), pp. 64–69; on buildings, see e.g. Angela Nicholls, *Almshouses in early modern England: charitable housing in the mixed economy of welfare, 1550–1725* (Boydell Press, Woodbridge, 2017).

67 Alexi Baker, ‘“Precision”, “perfection”, and the reality of British scientific instruments on the move during the 18th century’, *Material Cult. Rev.* **74–75**, 14–29 (2012); Richard Dunn, ‘Touching and cleaning: the routine work of an east London instrument supplier’, *Bull. Sci. Instr. Soc.* **89**, 21–26 (2006).

68 Corner, *op. cit.* (note 6), p. 24. On the 1679 fire, see Josten, *op. cit.* (note 51), p. 1643.

69 See e.g. Carla Bittel, Elaine Leong and Christine van Oertzen (eds), *Working with paper: gendered practices in the history of knowledge* (University of Pittsburgh Press, Pittsburgh, 2019).

70 Sara Pennell, ‘Invisible mending? Ceramic repair in eighteenth-century England’, in *The afterlife of used things* (ed. Ariane Fennetaux, Sophie Vasset and Amélie Junqua), pp. 107–121 (Routledge, New York, 2014).

71 *Ibid.*, p. 118.

72 Werrett, *op. cit.* (note 2), ch. 8. Sarah Easterby-Smith records similar changes in the case of plant collecting: Sarah Easterby-Smith, *Cultivating commerce: cultures of botany in Britain and France, 1760–1815* (Cambridge University Press, Cambridge, 2018), ch. 3.

